# Persistent perinatal protein malnutrition and post-weaning high-fat diet induce metabolic syndrome and hepatic UCP2 overexpression in IUGR offspring

**DOI:** 10.3389/fnut.2026.1905672

**Published:** 2026-08-31

**Authors:** Qiong Zeng, Yixi Wu, Yanting Chen, Qing Li, Zhijian Deng, Xiutong Chen, Jingsa Wang, Yiting He, Zeming Ma, Runjia Yang, Kun Lin

**Affiliations:** 1 Department of Neurology, The First Affiliated Hospital of Shantou University Medical College, Shantou, China; 2 Department of Endocrinology, The First Affiliated Hospital of Shantou University Medical College, Shantou, China; 3 Shantou University Medical College, Shantou, China

**Keywords:** developmental programming, intrauterine growth restriction, metabolic syndrome, non-alcoholic fatty liver disease, perinatal nutrition, uncoupling protein 2

## Abstract

**Introduction:**

Adults born with intrauterine growth restriction (IUGR) are at a significantly increased risk of developing metabolic syndrome. Postnatal nutritional patterns are critical modifiers of this risk. This study investigated the effects of different perinatal nutritional interventions on glucose/lipid metabolism and hepatic steatosis in IUGR offspring, and explored the role of hepatic uncoupling protein 2 (UCP2).

**Methods:**

An IUGR rat model was established via maternal low-protein (LP) diet during pregnancy. Male offspring were divided into five groups with distinct diets during gestation (G), lactation (L, 0-3w), and post-weaning (PW, 4-12w): ND+ND (Normal/ Normal/ Normal), ND+HFD (Normal/ High-fat/ High-fat), IUGR+ND (LP/ Normal/ Normal), IUGR+HFD (LP/ High-fat/ High-fat), and IUGR+LPD+HFD (LP/ LP/ High-fat).

**Results:**

At 12 weeks, only the IUGR+LPD+HFD group, exposed to persistent perinatal malnutrition followed by a high-fat diet, developed pronounced central obesity, glucose intolerance, insulin resistance, severe dyslipidemia (highest TG, lowest HDL-C), and marked hepatic steatosis. Notably, hepatic UCP2 protein expression was significantly upregulated specifically in the IUGR+LPD+HFD and ND+HFD groups, whereas UCP2 mRNA levels were unchanged across groups. In contrast, IUGR offspring receiving adequate postnatal nutrition (IUGR+ND, IUGR+HFD) were largely protected from these metabolic disturbances.

**Discussion:**

Our findings demonstrate that persistent protein restriction spanning both fetal and neonatal periods, followed by post-weaning high-fat intake, constitutes a critical”two-hit” model leading to full metabolic syndrome in IUGR offspring, which is closely associated with post-transcriptional upregulation of hepatic UCP2. This highlights the importance of early nutritional rehabilitation and suggests UCP2 as a potential mediator and biomarker of metabolically programmed risk.

## Background

Intrauterine growth restriction (IUGR) is a major perinatal challenge with significant long-term health implications. Robust epidemiological evidence links low birth weight, a proxy for IUGR, to an increased risk of obesity, type 2 diabetes, non-alcoholic fatty liver disease (NAFLD), and cardiovascular disease in adulthood (1, 2). This association forms the cornerstone of the Developmental Origins of Health and Disease (DOHaD) hypothesis, which posits that environmental cues during critical early-life windows can permanently “program” tissue structure and function, influencing disease susceptibility later in life (3, 4).

The liver, as the central metabolic organ, is a prime target for such programming. IUGR has been associated with reduced hepatocyte number, altered mitochondrial metabolism, and persistent changes in the expression of genes regulating glucose and lipid metabolism (5, 6). The postnatal nutritional environment plays a decisive role in modifying this programmed trajectory. While “catch-up growth” in IUGR infants was historically viewed as beneficial, it is now recognized that rapid weight gain, particularly when driven by excessive adiposity, is a strong predictor of later metabolic dysfunction (7, 8). The concept of a “two-hit” sequence-an initial fetal insult followed by postnatal nutritional excess-is central to understanding this heightened vulnerability (9). However, the specific impact of the timing and duration of early nutritional insults on shaping adult metabolic phenotypes in IUGR individuals remains incompletely understood.

Uncoupling protein 2 (UCP2) is a mitochondrial inner membrane protein that modulates energy balance and reactive oxygen species (ROS) production. It is expressed in key metabolic tissues including liver, adipose tissue, and pancreatic β-cells. UCP2 expression is linked to obesity, insulin resistance, and NAFLD pathogenesis (10, 11). Its regulation is influenced by dietary factors and metabolic stress (12). Intriguingly, early-life nutritional conditions may program UCP2 expression, positioning it as a potential molecular link between early environmental exposures and adult metabolic disease (13). Despite this, how different perinatal nutritional histories in IUGR models affect hepatic UCP2 and its relationship with specific metabolic outcomes is not well defined.

To address these gaps, we established a rat IUGR model via maternal protein restriction and designed a novel nutritional intervention group: IUGR + LPD + HFD, characterized by persistent protein undernutrition throughout both gestation and lactation, followed by a post-weaning high-fat diet. This model uniquely captures the effects of prolonged early deprivation followed by caloric excess. Our objectives were: (1) to systematically compare the effects of various perinatal-postnatal dietary regimens on the development of metabolic syndrome components in IUGR offspring; (2) to identify the critical nutritional windows that predispose to the most severe metabolic dysfunction; and (3) to investigate whether alterations in hepatic UCP2 expression are associated with distinct metabolic programming phenotypes.

## Materials and methods

### Animals and dietary interventions

All experimental procedures were approved by the Animal Ethics Committee of Shantou University Medical College and conducted in accordance with the Declaration of Helsinki. Timed-pregnant Sprague–Dawley rats were randomly assigned to either a normal-protein diet (N, 21% protein by energy) or an isocaloric low-protein diet (L, 8% protein by energy) throughout gestation. To prolong the window of protein deprivation, the LP diet was deliberately continued during lactation exclusively in the IUGR + LPD + HFD group, whereas all other IUGR groups received nutritional rehabilitation during this period. After delivery, male pups from L dams with a birth weight more than two standard deviations below the mean of the N group were identified as IUGR offspring. Only male offspring were used to avoid confounding effects of female sex hormones.

Lactating dams and their litters were maintained on specified diets. At weaning (postnatal day 21), male offspring were housed individually and continued on their assigned diets until 12 weeks of age. Five experimental groups were generated: (1) ND + ND (*n* = 8): Control. Offspring from N dams, fed normal diet during lactation and post-weaning; (2) ND + HFD (*n* = 6): Postnatal overnutrition. Offspring from N dams, fed high-fat diet (HFD) during lactation and post-weaning; (3) IUGR + ND (*n* = 6): IUGR with postnatal rehabilitation. IUGR offspring, dams fed normal diet during lactation, offspring fed normal diet post-weaning; (4) IUGR + HFD (*n* = 6): IUGR with postnatal HFD. IUGR offspring, dams fed HFD during lactation, offspring fed HFD post-weaning; (5) IUGR + LPD + HFD (*n* = 5): Persistent perinatal malnutrition + HFD. IUGR offspring, dams continued on LP diet during lactation, offspring switched to HFD post-weaning. Detailed diet compositions are provided in Supplementary Table S1; the HFD contained lard, sesame oil, peanut kernel, powdered milk, and yolk powder as major fat sources, with an estimated fatty acid composition of approximately 35–40% saturated, 40–45% monounsaturated, and 15–20% polyunsaturated fatty acids. Body weight was monitored weekly.

All metabolic and histologic analyses were performed at 12 weeks of age, unless otherwise specified. The time point of postnatal day 21 (weaning) served only as the starting point for post-weaning dietary interventions and group allocation, not as an endpoint for metabolic assessment.

### Metabolic tests at 12 weeks

Oral Glucose Tolerance Test (OGTT) and Insulin Release Test (IRT): After an overnight fast, baseline blood samples were taken from the tail vein (0 min). Rats were then orally gavaged with a 20% glucose solution (2 g/kg body weight). Blood glucose (using a glucometer) and plasma insulin (using a rat ELISA kit) were measured at 30, 60, and 120 min post-gavage. Areas under the curve for glucose (AUCglucose) and insulin (AUCinsulin) were calculated.

Insulin Tolerance Test (ITT): After a 6-h fast, baseline blood glucose was measured. Human insulin (0.5 U/kg) was injected intraperitoneally, and blood glucose was measured at 30, 60, and 90 min. The glucose reduction rate was calculated.

### Sample collection and biochemical analysis

After metabolic tests, rats were anesthetized. Body weight and naso-anal length were measured to calculate Lee’s index (body weight^1^/^3^/length × 1,000). Blood was collected via cardiac puncture; serum was separated for analysis of total cholesterol (TC), triglycerides (TG), high-density lipoprotein cholesterol (HDL-C), and low-density lipoprotein cholesterol (LDL-C) using an automated biochemical analyzer.

Liver and visceral (perirenal/epididymal) fat pads were dissected and weighed. A portion of the liver was fixed in 4% paraformaldehyde for histology; another portion was snap-frozen for RNA extraction.

### Histological and molecular analyses

Liver Histology: For each animal, a single liver tissue block from the same lobe was fixed in 4% paraformaldehyde, embedded in paraffin, and serially sectioned at 4 μm thickness. Three sections per animal were stained with Hematoxylin & Eosin (H&E) for general morphology. For Oil Red O staining, three frozen sections per animal were used to visualize neutral lipid droplets. Steatosis was assessed in a blinded manner by two independent observers.

Immunohistochemistry (IHC) for UCP2: Three serial sections per animal were processed for IHC using a specific anti-UCP2 primary antibody, followed by appropriate secondary antibodies and DAB development. Negative controls omitted the primary antibody. Five random high-power fields (400×) per section were captured. The average optical density of positive staining was quantified using Image-Pro Plus 6.0 software, and the mean value per animal was used for group comparisons.

Quantitative Real-Time PCR (qPCR): Total RNA was extracted from frozen liver tissue. cDNA was synthesized, and qPCR was performed using SYBR Green chemistry on an ABI Prism 7,500 system. UCP2 mRNA levels were normalized to β-actin and expressed relative to the ND + ND group using the 2^−ΔΔCt^ method. Primer sequences: UCP2 forward: 5′-CCCAATGTTGCCCGAAATG-3′, reverse: 5′-GCAATGACGTGGTGCAGA-3′.

### Statistical analysis

Data are presented as mean ± SEM. Statistical analyses were performed using SPSS 19.0. Differences among groups were assessed by one-way ANOVA followed by LSD post-hoc test for multiple comparisons. A *p* value < 0.05 was considered statistically significant.

## Results

### Growth patterns and body composition

**Figure 1:**
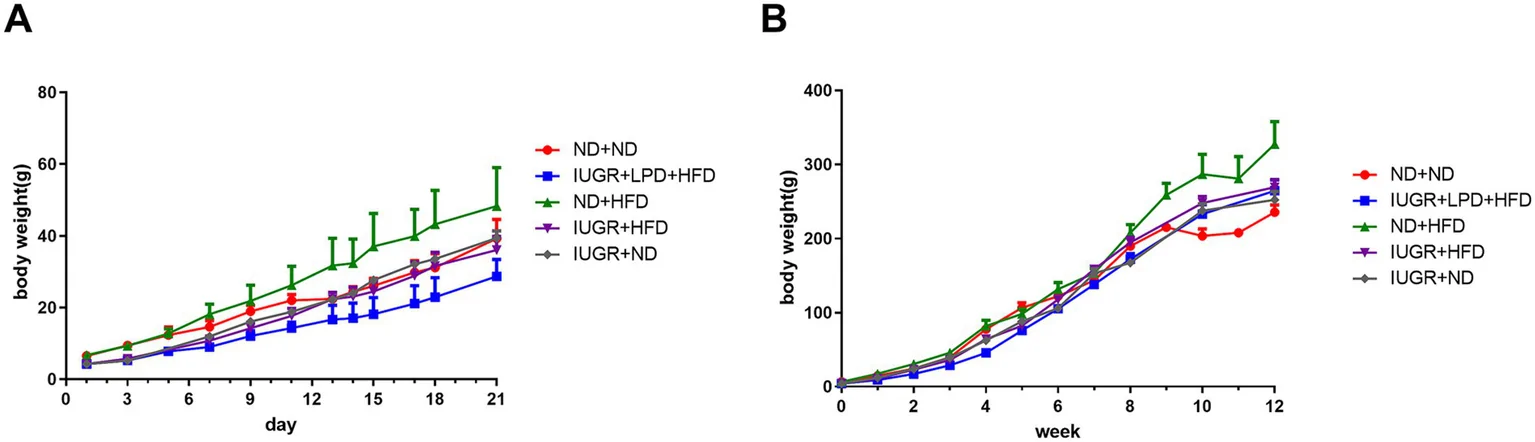
Growth curves of rats in different experimental groups. **(A)** Body weight during the lactation period (0–21 days of age). **(B)** Body weight from birth to 12 weeks of age. Values are mean ± SEM. ND, normal diet; HFD, high-fat diet; LPD, low-protein diet (malnutrition).

During lactation, pups in the ND + HFD group gained weight faster than controls, while growth was significantly slower in the IUGR + LPD + HFD group (*p* < 0.05, Figure 1A). After weaning, all IUGR groups exhibited catch-up growth, achieving body weights comparable to or exceeding the ND + ND group by week 10 (Figure 1B). At 12 weeks, the ND + HFD group had the highest absolute body weight.

All IUGR groups had a higher Lee’s index than the ND + ND group, indicating increased adiposity (Table 1). However, only the IUGR + LPD + HFD group developed marked central obesity, evidenced by a significantly greater wet weight of visceral fat pads and a higher visceral fat-to-body weight ratio compared to the other IUGR groups (*p* < 0.05). Interestingly, the liver-to-body weight ratio was significantly lower in the IUGR + ND and IUGR + HFD groups compared to both the ND + ND and IUGR + LPD + HFD groups (*p* < 0.05).

**Table 1:** Weight, length, waist, weight of peritoneal fat in different group of rats.

Groups	*n*	Birth weight (g)	Weight at 12 weeks old (g)	Length (cm)	Waist (cm)	Lee’s index	Wet weight of peritoneal adipose tissue (g)	Unit weight of adipose (×10-3%)	Wet weight of liver (g)	The ratio of wet weight per unit weight of liver (g/100 g)
ND + ND	8	6.52 ± 0.50	252.88 ± 22.90	22.3 ± 2.7	14.8 ± 0.6	285.87 ± 27.78	2.70 ± 0.56	10.65 ± 1.84	8.48 ± 0.89	3.36 ± 0.32
ND + HFD	6	6.72 ± 0.60	351.83 ± 84.41^a^	21.4 ± 1.5	18.0 ± 2.1^aa^	327.17 ± 4,42^aa^	10.56 ± 6.63^aa^	27.97 ± 11.94^aa^	10.37 ± 3.43	2.89 ± 0.31^ab^
IUGR + ND	6	4.24 ± 0.19^aa^	282.00 ± 32.43	20.8 ± 1.7	17.6 ± 1.2^a^	314.80 ± 6.43^aa^	4.13 ± 1.13	14.65 ± 3.81	8.10 ± 1.57	2.86 ± 0.29^ab^
IUGR + HFD	6	4.23 ± 0.42^aa^	285.12 ± 27.58	20.8 ± 1.0	16.1 ± 0.6^a^	314.96 ± 4.50^aa^	3.93 ± 1.29	13.54 ± 3.07	8.66 ± 0.89	3.05 ± 0.20^ab^
IUGR + LPD + HFD	5	4.24 ± 0.40^aa^	274.80 ± 30.14	20.6 ± 0.9^a^	17.7 ± 1.2^aa^	315.93 ± 4.42^aa^	6.16 ± 2.05	22.00 ± 5.11^aa^	9.51 ± 1.21	3.46 ± 0.22

Compared with the ND + ND group, ^a^*p* < 0.05; ^aa^*p* < 0.01. Compared with the IUGR + LPD + HFD group; ^b^*p* < 0.05.

### Glucose metabolism and insulin sensitivity

**Figure 2:**
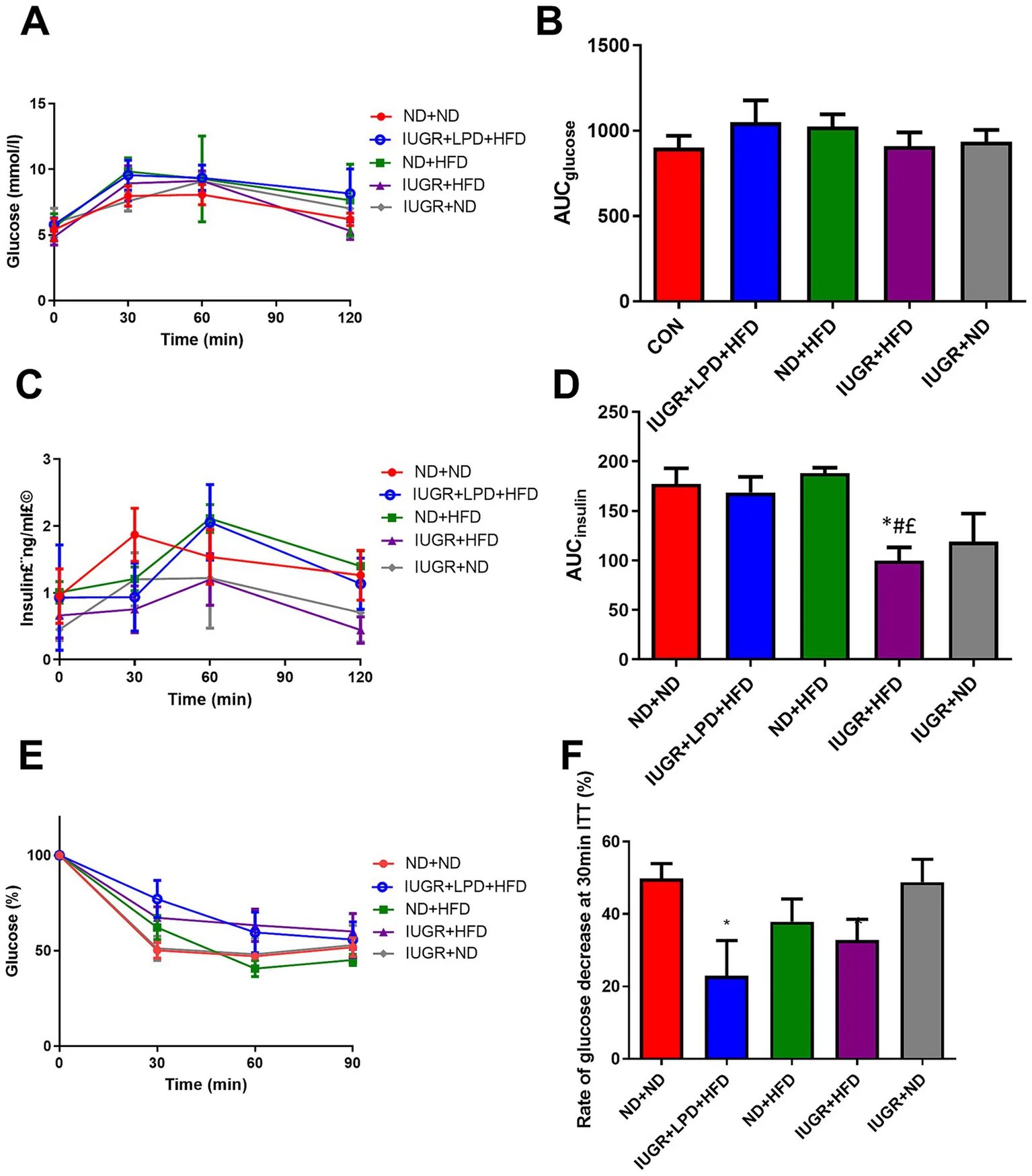
OGTT, IRT and ITT in different groups of rats. **(A)** Glucose levels in 120 min OGTT; **(B)** Area under the curve (AUC) of glucose in OGTT; **(C)** Insulin levels in 120 min IRT; **(D)** AUCinsulin in IRT; **(E)** Rate of glucose reduction in 90 min ITT; **(F)** Rate of glucose reduction at 30 min ITT. *, compared with the ND + ND group, *p* < 0.05#, compared with the IUGR + LPD + HFD group, *p* < 0.05; £, compared with the ND + HFD group, *p* < 0.05. OGTT ral glucose tolerance test; IRT nsulin release test; ITT nsulin tolerance test. Group abbreviations refer to “Method” section.

Fasting blood glucose levels were similar across groups. During the OGTT, blood glucose levels at 30 min and the AUCglucose were significantly elevated in the IUGR + LPD + HFD and ND + HFD groups compared to the ND + ND group (*p* < 0.05), indicating impaired glucose tolerance (Figures 2A,B). The IUGR + ND and IUGR + HFD groups had normal glucose excursions.

Plasma insulin response during the OGTT (IRT) revealed distinct patterns (Figures 2C,D). The IUGR + ND and IUGR + HFD groups showed a blunted and delayed insulin secretory response, with a significantly lower AUCinsulin than the ND + ND group (*p* < 0.05). In contrast, the IUGR + LPD + HFD group exhibited a sluggish rise but eventually reached a high late-phase insulin level. The ITT further supported the presence of insulin resistance in the IUGR + LPD + HFD group, which consistently showed the slowest rate of blood glucose decline across the 90-min time course (*p* < 0.05 vs. ND + ND, Figure 2E), with the most pronounced difference observed at 30 min (Figure 2F). The ND + HFD group also displayed reduced insulin sensitivity.

### Serum lipid profile and hepatic steatosis

The IUGR + LPD + HFD group exhibited the most adverse lipid profile (Table 2). It had the highest serum TG level and the lowest HDL-C level, resulting in a TG/HDL-C ratio that was dramatically higher than all other groups (*p* < 0.01). The ND + HFD group showed a moderate, non-significant worsening of lipids.

**Table 2:** Fasting serum lipid levels in different groups of rats.

Groups	*n*	TC (mmol/L)	TG (mmol/L)	LDL (mmol/L)	HDL (mmol/L)	TG/HDL-C ratio
ND + ND	8	1.39 ± 0.61	0.39 ± 0.10^b^	0.64 ± 0.22	0.69 ± 0.18^b^	0.56 ± 0.10^b^
ND + HFD	6	1.57 ± 0.11	0.47 ± 0.16	0.64 ± 0.37	0.62 ± 0.13^b^	0.76 ± 0.11^b^
IUGR + ND	6	1.72 ± 0.26	0.44 ± 0.82^b^	0.88 ± 0.23	0.66 ± 0.12^b^	0.68 ± 0.22^b^
IUGR + HFD	6	1.58 ± 0.13	0.40 ± 0.13^b^	0.83 ± 0.09	0.58 ± 0.14^b^	0.69 ± 0.14^b^
IUGR + LPD + HFD	5	1.41 ± 0.39	0.78 ± 0.62	0.71 ± 0.19	0.39 ± 0.13	1.86 ± 1.05

^b^Compared with the IUGR + LPD + HFD group, *p* < 0.05.

**Figure 3:**
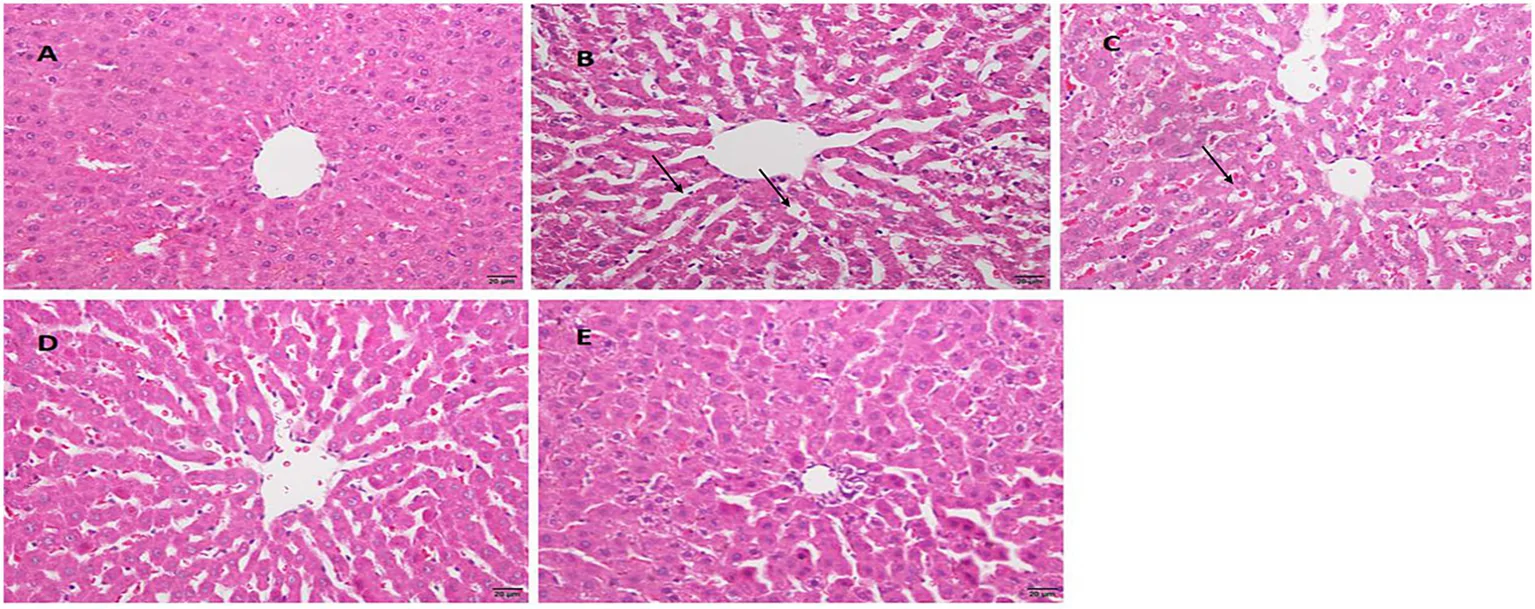
HE staining of liver tissue in different groups of rats (×400): **(A)** ND + ND group; **(B)** IUGR + LPD + HFD group; **(C)** ND + HFD group; **(D)** IUGR + HFD group; **(E)** IUGR + ND group. Arrows indicate lipid vacuoles and sinusoidal dilation **(B, C)**.

**Figure 4:**
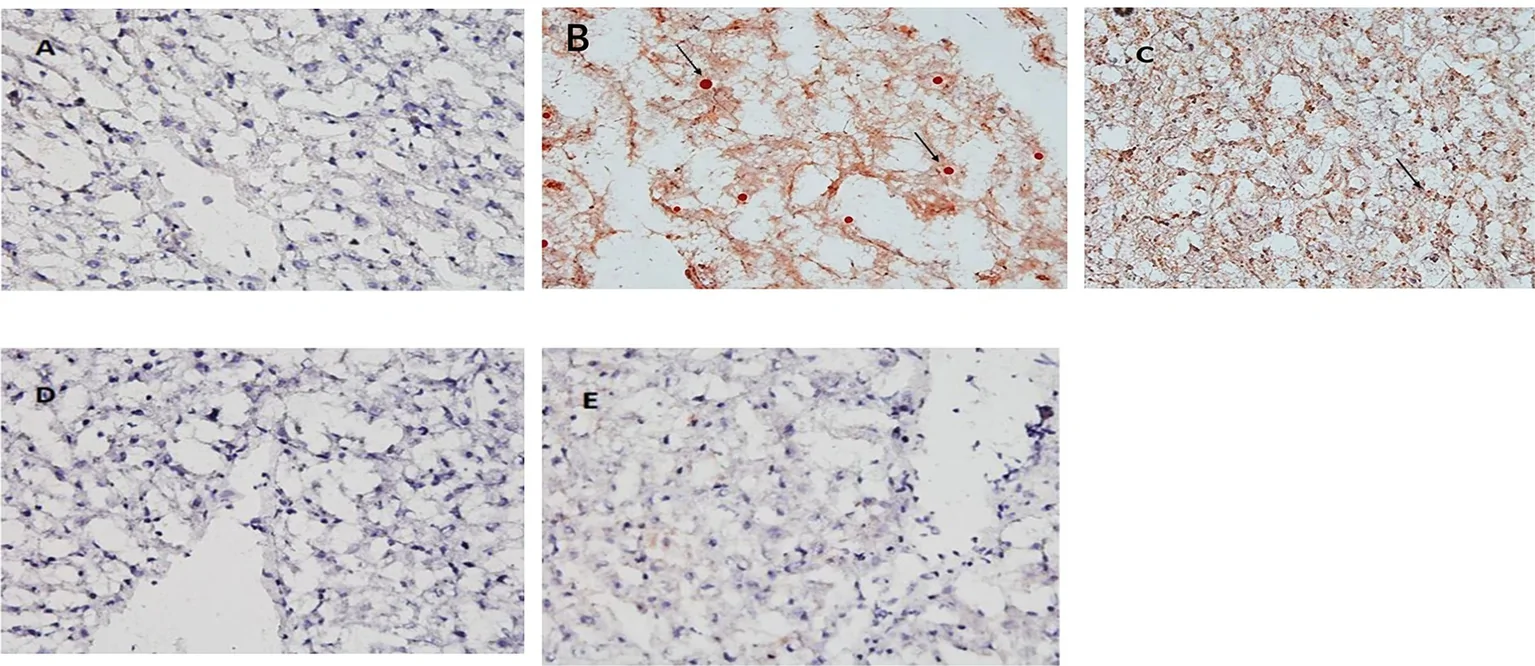
Oil Red O staining of liver tissue in different groups of rats. (×400) **(A)** ND + ND group; **(B)** IUGR + LPD + HFD group; **(C)** ND + HFD group; **(D)** IUGR + HFD group; **(E)** IUGR + ND group. Arrows indicate Oil Red O-positive lipid droplets **(B, C)**.

H&E staining revealed normal liver architecture in the ND + ND, IUGR + ND, and IUGR + HFD groups (Figures 3A,D,E). The ND + HFD group showed mild hepatic steatosis with sinusoidal dilation (Figure 3C). The IUGR + LPD + HFD group displayed severe macrovesicular steatosis, with numerous large lipid vacuoles within hepatocytes (Figure 3B). Oil Red O staining confirmed these findings: intense red lipid droplets were abundant in the livers of the IUGR + LPD + HFD and ND + HFD groups, with the former being the most severe, while minimal staining was observed in the other groups (Figure 4).

### Hepatic UCP2 protein and mRNA expression

**Figure 5:**
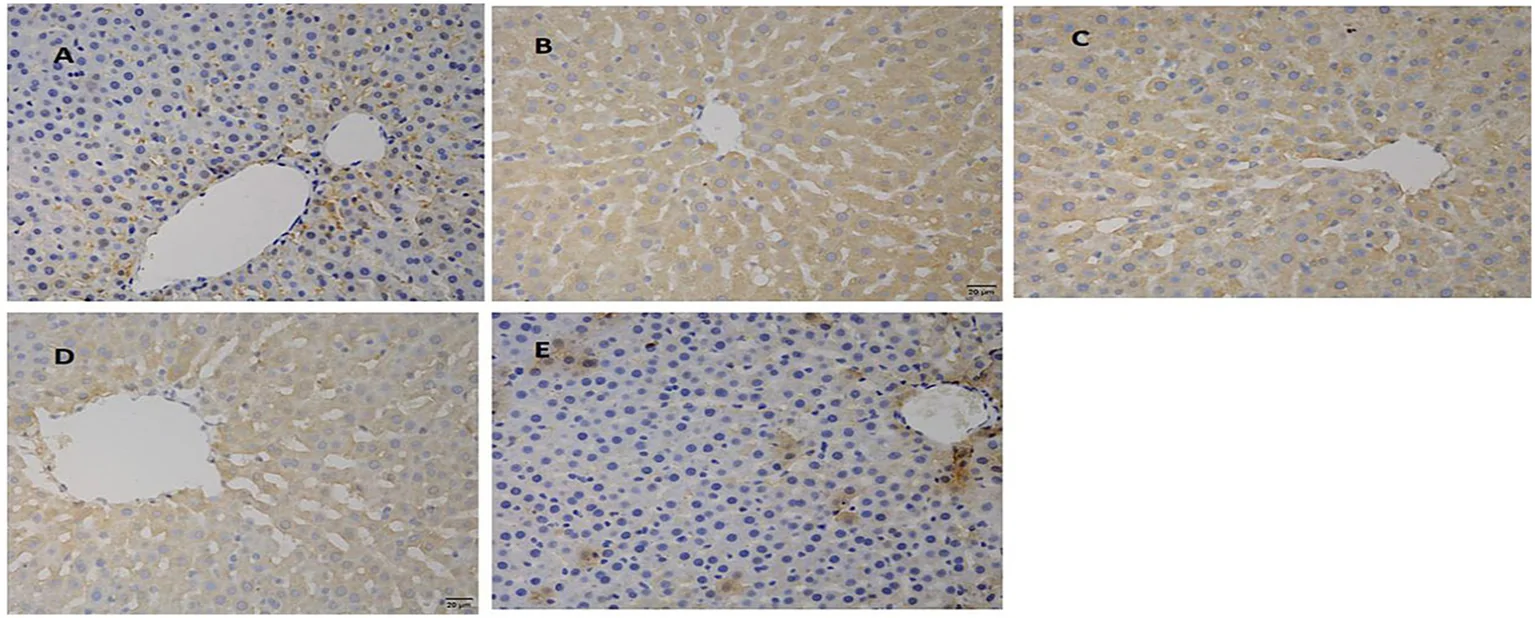
Immunohistochemistry of UCP2 in liver of rats in different groups. (×400) **(A)** ND + ND group; **(B)** IUGR + LPD + HFD group; **(C)** ND + HFD group; **(D)** IUGR + HFD group; **(E)** IUGR + ND group.

**Figure 6:**
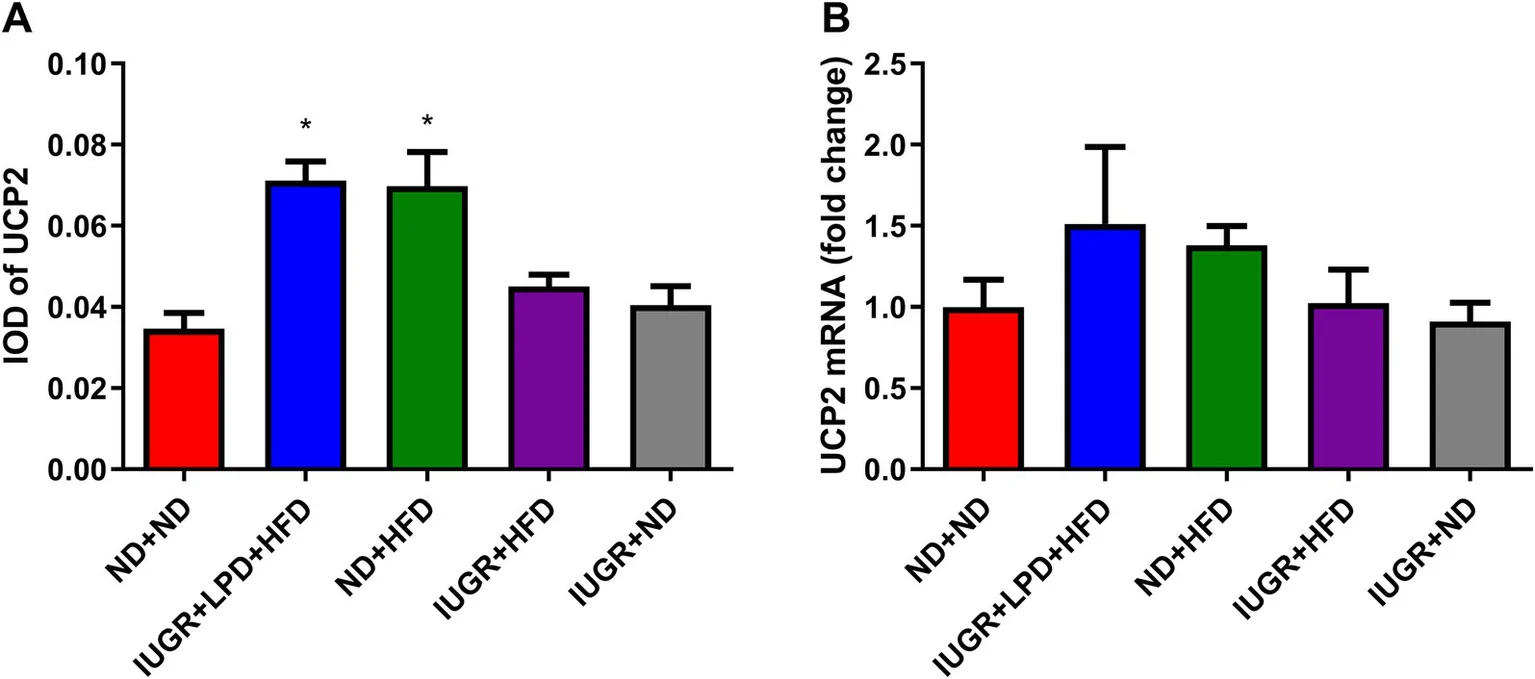
The average optical density results of UCP2 immunohistochemistry **(A)** and the expression of UCP2 mRNA **(B)** in liver of rats in different groups. *, compared with the ND + ND group, *p* < 0.05.

IHC analysis demonstrated basal UCP2 protein expression in the livers of the ND + ND, IUGR + ND, and IUGR + HFD groups (Figures 5A,D,E). In contrast, significantly stronger UCP2 immunopositivity was observed in hepatocytes from the IUGR + LPD + HFD and ND + HFD groups (Figures 5B,C). Quantitative analysis of IHC optical density confirmed a significant, approximately 2-fold increase in UCP2 protein levels in these two groups compared to the ND + ND group (*p* < 0.05, Figure 6A). Remarkably, qPCR analysis revealed no significant differences in hepatic UCP2 mRNA abundance among any of the five groups (Figure 6B), indicating post-transcriptional regulation of UCP2 protein expression.

## Discussion

This study introduces a novel nutritional model (IUGR + LPD + HFD) that recapitulates a high-risk early-life trajectory: persistent undernutrition from conception through the suckling period, followed by exposure to an obesogenic diet. Our key finding is that this specific “two-hit” sequence programs a full spectrum of metabolic syndrome components-central obesity, glucose intolerance, insulin resistance, atherogenic dyslipidemia, and severe NAFLD-in young adult IUGR offspring. Crucially, providing adequate nutrition during the lactation period, even after fetal protein restriction, conferred substantial protection against these adverse outcomes. This underscores the lactation period as a critical and independent window for nutritional rescue of metabolically programmed risk, challenging the view that fetal programming is irreversible and highlighting the importance of developmental stage-specific interventions (14).

Collectively, these findings demonstrate that metabolic programming induced by fetal protein restriction is not irreversible; timely nutritional rehabilitation during lactation can effectively attenuate the programmed risk, even when offspring are subsequently exposed to an obesogenic diet. The severity of metabolic dysfunction in the IUGR + LPD + HFD group aligns with and extends the “thrifty phenotype” hypothesis (15). We propose that the duration of the initial nutritional insult is paramount. Continuous protein restriction throughout gestation and lactation likely deepens the metabolic and endocrine adaptations for survival, creating a more inflexible “thrifty” phenotype characterized by profound alterations in hepatic development and systemic metabolic setpoints (16). This interpretation is supported by the comparison between the IUGR + HFD and IUGR + LPD + HFD groups: although both were exposed to an identical post-weaning HFD, only the latter—which sustained protein restriction through lactation—developed the full metabolic syndrome, confirming that the duration of early protein deprivation, rather than HFD alone, determines the severity of programming. When this is followed by a high-energy diet, the capacity for metabolic flexibility is overwhelmed, leading to rapid decompensation. In contrast, rehabilitation during lactation (IUGR + ND/HFD) may allow partial recalibration of developmental pathways, particularly in the liver and adipose tissue, thereby enhancing metabolic resilience (6). Notably, the IUGR + ND group, despite sharing the same low birth weight as the IUGR + LPD + HFD group, was largely protected from metabolic disturbances, indicating that postnatal nutritional rehabilitation during lactation can override the programming effects of fetal undernutrition. This is consistent with human studies suggesting that the pattern, not just the fact, of postnatal catch-up growth influences metabolic risk (17). The observed protection in the IUGR + HFD group is particularly instructive, suggesting that adequate nutrition during lactation can build resilience that partially buffers against a later obesogenic challenge, a concept with significant translational implications (18).

Of note, in rodents, the neonatal (pre-weaning) period corresponds developmentally to the third trimester of human pregnancy, a critical window for organ maturation and metabolic programming in IUGR offspring. This cross-species temporal correspondence further explains why extending protein restriction through lactation—the rodent equivalent of the human third trimester—produced the most severe phenotype in our model, whereas restriction limited to gestation alone was insufficient to induce the full metabolic syndrome. These findings highlight the translational relevance of our model to human IUGR, in which the third trimester is recognized as a particularly vulnerable window for the programming of adult metabolic health.

A pivotal and novel observation is the specific upregulation of hepatic UCP2 protein exclusively in the metabolically compromised groups (IUGR + LPD + HFD and ND + HFD). This upregulation was dissociated from mRNA levels, implicating robust post-transcriptional control. This mechanism may involve relief of translational repression mediated by upstream open reading frames in the UCP2 mRNA, a process known to be activated by cellular stressors such as elevated fatty acids or ROS (19). Importantly, UCP2 upregulation was not a mere consequence of IUGR per se, as it was absent in the metabolically protected IUGR groups. This positions UCP2 not as a simple marker of IUGR, but as a dynamic molecular sensor and potential effector of metabolically stressful environments arising from nutrient mismatch-particularly the combination of early deprivation and later excess (8).

The functional implications of increased hepatic UCP2 are complex and may represent a double-edged sword. On one hand, UCP2-mediated mild mitochondrial uncoupling can serve an adaptive role by attenuating excessive ROS production driven by lipid overload, thus mitigating oxidative damage (20). On the other hand, this uncoupling action reduces the efficiency of oxidative phosphorylation and ATP synthesis. In the energy-demanding context of a fatty liver, this energy deficit could have deleterious consequences: impairing VLDL assembly and export, promoting *de novo* lipogenesis through altered cellular energy sensing (e.g., suppressed AMPK activity), and impairing hepatic insulin signaling, including enhanced gluconeogenesis, thus exacerbating hepatic insulin resistance (11, 21–23). Thus, we postulate a vicious cycle: lipid overload and stress trigger UCP2 upregulation, which in turn compromises hepatic energy metabolism, further aggravating lipid accumulation and insulin resistance, thereby generating more cellular stress (10, 24). This cycle may be central to the rapid progression from simple steatosis to metabolic dysfunction in our high-risk model.

Our study has several limitations that point to future directions. First, the use of only male offspring precludes understanding of sex-specific responses to early nutritional programming; whether similar programming effects occur in females remains to be determined. Second, assessment at a young adult age leaves the long-term trajectory and potential emergence of later comorbidities unknown. Third, while we establish a strong association, the causal role of UCP2 in driving the observed phenotype remains to be determined. Future studies employing liver-specific UCP2 knockout or overexpression in this model are warranted. Fourth, the LP and control diets were not matched for methionine content, as the LP diet used casein as the primary protein source whereas the control diet contained soybean powder and fish meal; therefore, the observed programming effects may involve methionine/methyl donor availability and associated epigenetic mechanisms, rather than protein deficiency alone. Future studies incorporating methionine supplementation would help dissect the specific contribution of each. Furthermore, measuring direct indicators of mitochondrial function (respiratory capacity, ATP levels), oxidative stress, and inflammation would provide mechanistic depth. Finally, exploring the upstream signals (e.g., specific fatty acid species, endoplasmic reticulum stress) and potential epigenetic regulators of UCP2 expression could unveil novel layers of metabolic programming.

## Conclusion

We demonstrate that persistent protein malnutrition spanning the fetal and neonatal periods, followed by post-weaning high-fat feeding, creates a detrimental “two-hit” nutritional milieu that programs severe metabolic syndrome in IUGR offspring. This metabolic deterioration is specifically associated with post-transcriptional upregulation of hepatic UCP2 protein, identifying it as a novel hallmark of this high-risk programming phenotype. In contrast, ensuring adequate nutrition during the critical window of lactation can markedly attenuate these programmed risks. These findings emphasize the profound importance of early-life nutritional quality and timing for the long-term metabolic health of growth-restricted individuals. Hepatic UCP2 emerges not only as a promising biomarker but also as a potential mechanistic node and therapeutic target for breaking the cycle of metabolically programmed disease.

## Data Availability

The raw data supporting the conclusions of this article will be made available by the authors, without undue reservation.

## Glossary

IUGR: Intrauterine Growth Restriction
UCP2: Uncoupling Protein 2
NAFLD: Non-alcoholic Fatty Liver Disease
DOHaD: Developmental Origins of Health and Disease
LP: Low-Protein (diet)
N: Normal-protein (diet)
L: Low-protein (diet)
PW: Post-weaning
ND: Normal Diet
HFD: High-Fat Diet
LPD: Low-Protein Diet
TG: Triglycerides
HDL-C: High-Density Lipoprotein Cholesterol
OGTT: Oral Glucose Tolerance Test
IRT: Insulin Release Test
AUC: Area Under the Curve
ITT: Insulin Tolerance Test
TC: Total Cholesterol
LDL-C: Low-Density Lipoprotein Cholesterol
H&E: Hematoxylin & Eosin
IHC: Immunohistochemistry
qPCR: Quantitative Real-Time Polymerase Chain Reaction
SEM: Standard Error of the Mean
ANOVA: Analysis of Variance
LSD: Least Significant Difference
ROS: Reactive Oxygen Species
ATP: Adenosine Triphosphate
VLDL: Very Low-Density Lipoprotein
