# Supplementary material for: Persistent perinatal protein malnutrition and post-weaning high-fat diet induce metabolic syndrome and hepatic UCP2 overexpression in IUGR offspring
Source: Front Nutr. 2026 Aug 31;13:1905672. doi: 10.3389/fnut.2026.1905672 (PMC13572690; doi:10.3389/fnut.2026.1905672)
Supplement: Supplementary file 1 [file Table_1.DOCX]

|  | normal chow | |  | 8% Protein chow | |  | High-fat chow | |  |
| --- | --- | --- | --- | --- | --- | --- | --- | --- | --- |
| Component | g% | kcal% | | g% | kcal% | | g% | kcal% | |
| Protein | 21 | 23.5 | | 8 | 8.2 | | 17 | 14 | |
| Fat | 6 | 15.1 | | 5 | 11.6 | | 27 | 49.9 | |
| Carbohydrates | 55 | 61.4 | | 78.1 | 80.2 | | 44 | 36.1 | |
| Total |  | 100 | |  | 100 | |  | 100 | |
| Energy (kcal/g) | 3.58 |  | | 3.894 |  | | 4.87 |  | |
|  |  |  | |  |  | |  |  | |
| Ingredient | g/1000g | | | g/1000g | | | g/1000g | | |
| Flour | 0 | | | 0 | | | 125 | | |
| Soybean powder | 204.3 | | | 0 | | | 87.5 | | |
| Wheat middlings | 0 | | | 0 | | | 66.5 | | |
| Fish meal | 71.5 | | | 0 | | | 21 | | |
| Dicalcium phosphate | 13.3 | | | 0 | | | 10 | | |
| Calcium carbonate | 5.6 | | | 0 | | | 6.5 | | |
| Additive | 0 | | | 0 | | | 2.5 | | |
| Choline chloride | 2.6 | | | 0 | | | 1.5 | | |
| Casein protein | 0 | | | 72 | | | 0 | | |
| Corn starch | 408.5 | | | 411 | | | 170.5 | | |
| Maltodextrin | 224.7 | | | 170 | | | 0 | | |
| Sucrose | 0 | | | 200 | | | 0 | | |
| Cellulose | 0 | | | 50 | | | 0 | | |
| Soybean oil | 4.6 | | | 50 | | | 9 | | |
| Premix | 0 | | | 47 | | | 0 | | |
| Mineral mix | 13.8 | | | 0 | | | 0 | | |
| Vitamin mix | 10.2 | | | 0 | | | 0 | | |
| Yeast | 40.9 | | | 0 | | | 0 | | |
| Lard | 0 | | | 0 | | | 150 | | |
| Sucrose | 0 | | | 0 | | | 100 | | |
| Powdered milk | 0 | | | | | | 50 | | |
| Yolk powder | 0 | | | 0 | | | 100 | | |
| Sesame oil | 0 | | | 0 | | | 50 | | |
| Peanut kerne | 0 | | | 0 | | | 50 | | |

S1 Table. chow composition of each group
